# Sex Differences in Lung Immunity

**DOI:** 10.1111/imr.70102

**Published:** 2026-02-15

**Authors:** Franz Puttur, Clare M. Lloyd

**Affiliations:** ^1^ National Heart and Lung Institute Imperial College London London UK

**Keywords:** aging, allergy, biological sex, inflammation, lung, processes, sex hormones, tissues

## Abstract

Biological sex has a significant impact on how the immune system develops and responds to foreign and self‐antigens. Sex differences exist in innate and adaptive immune cells, both at homeostasis and in the context of infection and inflammatory diseases such as asthma, cancer, and autoimmune disorders. Women generate stronger immune responses and are more susceptible to developing autoimmune conditions, while males are more prone to acute viral infections and developing certain cancers. Some immunological differences persist throughout life, while others emerge only after puberty and before reproductive senescence. Additionally, environmental exposures can affect the influence of biological sex in regulating immune function. This is particularly pertinent at mucosal surfaces such as the lungs, where lung immune defenses are constantly exposed to foreign material during respiration. Consequently, environmental factors together with genetics, age and sex hormones play a vital role in governing lung tissue immune responses between the sexes. In this context, we highlight studies that support the need for considering sex as an important biological variable in lung immunological research. This knowledge will provide a benchmark for understanding sex‐driven immunological mechanisms that underpin disease development and may inform new avenues targeted for generating sex‐specific therapies in lung disease.

## 1 Introduction

Tissue immunity requires an elaborate dialogue between infiltrating and tissue‐resident immune cells, together with the local structural and stromal cells that comprise the organ structure. These complex cellular interactions take place in the context of the surrounding tissue matrix, together with the lipids and proteins secreted by these cells, which collaboratively influence and fine‐tune these biological interactions. Moreover, cell–cell and cell‐protein interactions are exquisitely regulated by a multitude of intrinsic and extrinsic factors, which calibrate the tone and balance the magnitude of the immune response. Although these factors include age, diet and circadian rhythms, one of the most fundamental factors is that of biological sex. The influence of sex on tissue immunity is clear, as many inflammatory diseases exhibit a distinct sex bias in both incidence and pathophysiology. The impact of sex on the development of immune dysfunction may occur as a result of the expression of chromosome‐encoded genes, but also due to sex steroid hormone signalling. This is a vital consideration due to the fluctuating temporal levels of sex hormones, which may occur broadly across the life course, coinciding with the key stages of reproductive life, but also on a more frequent, regular basis, such as monthly during menstruation and daily according to circadian rhythms.

Lung diseases are common worldwide, encompassing asthma, chronic obstructive pulmonary disease (COPD) and interstitial lung diseases (ILD) as well as genetic diseases such as cystic fibrosis (CF). All of these diseases involve some degree of fundamental immune dysregulation, which contributes to the development of pathophysiology. Thus, the influence of sex on the lung immune system and specific immune cells may contribute to the differing phenotypes of lung disease between men and women. In addition, it is now recognized that there may be fundamental underlying differences in lung structure that also impact on pulmonary health and disease development. We will discuss these differences in lung structural and immune cells, their impact on the phenotypes and functional outcomes in common respiratory diseases and infections, as well as interrogate the environmental factors that calibrate the degree of sex‐specific regulation of lung immunity.

### 1.1 Sex Differences in Foetal and Early‐Life Lung Development

**FIGURE 1:**
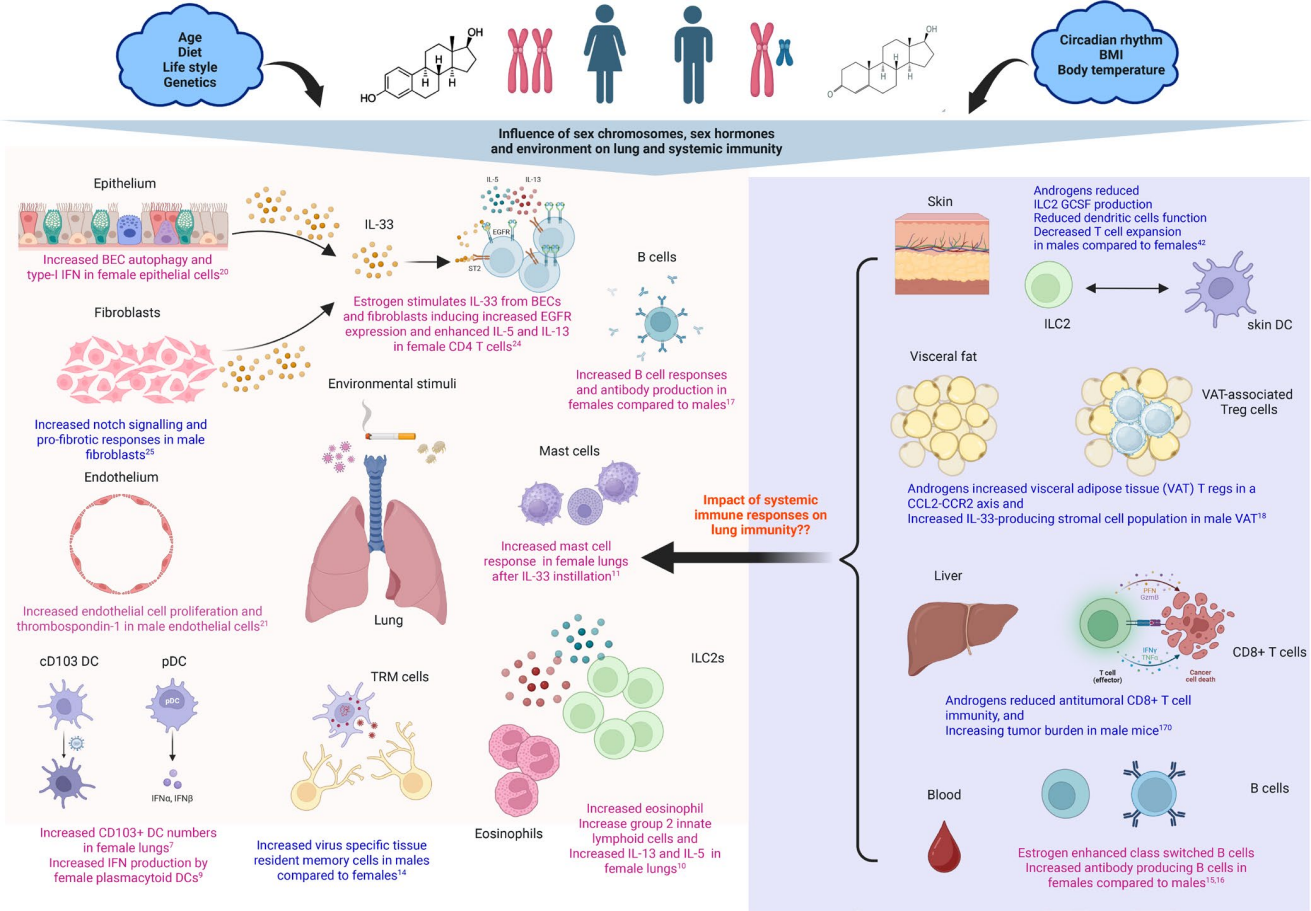
**Influence of sex chromosomes, sex hormones and the environment on lung immunity**. Shown here is how sex hormones and sex chromosomes influence local lung immune responses by impacting individual lung structural cells and immune cells differently between females (magenta) and males (blue). For example, Androgens enhance genes associated with pro‐fibrotic responses in male fibroblasts, while estrogens enhance type‐2 immune responses in asthma, enhancing group‐2 innate lymphoid cell and eosinophil activity and increasing type 2 cytokines in females. Importantly, sex hormones can also influence systemic immune responses, which in turn can impact lung immunity. These sex hormone‐immune interactions are intertwined with environmental influences such as age, diet, circadian rhythm, microbiome, body temperature and lifestyle, increasing the complexity of understanding the specific contribution of biological sex in lung immunity. Created with https://BioRender.com.

The respiratory system harbors remarkable anatomical and physiological sex differences that profoundly shape lung development and severity of lung diseases throughout the life course. Indeed, sex differences in lung development commence from the prenatal period. In humans, after 16 weeks of gestation, the lungs of males and females begin to diverge significantly [1]. Intrinsically, female airways have lower specific airway resistance than males and increased surfactant production in the alveolar lining, surfactant production being essential for alveolar formation and stability, reducing surface tension and facilitating airflow [2]. Sex hormones strongly contribute to these differences in lung maturation, whereby androgens suppress the surfactant lipid production, while estrogens promote surfactant production and neonatal alveologenesis [3]. Therefore, premature neonatal males are at a greater risk of respiratory distress syndrome‐associated morbidity [4].

Three‐dimensional morphometric analysis of computed tomography scans of lungs [1] suggests that although men have larger lungs than women, the alveolar number and alveolar dimensions and elasticity of lung parenchyma do not differ between the sexes [1, 5]. These early‐life differences in lung capacity predict later lung function which results in distinctive developmental trajectories between females and males. These sex‐associated differences are further influenced by varying levels of individual sex hormones throughout different stages of development, underscoring the importance of interrogating sex‐specific differences in lung function and susceptibility to lung pathologies throughout the life course.

### 1.2 Sex Differences in the Lung Immune Profile (Figure 1)

Biological sex strongly contributes to differences in the immune landscape, including varying phenotypes of immune cells and differences between circulating and tissue‐resident immune cell subsets. This is reflected in both the innate and adaptive arms of the immune system. A high‐throughput phenotyping study of over 500 genetic knockout mouse strains identified biological sex as the primary contributor to variability in immune cell populations [6].

Recent studies by Belios et al. showed that, at homeostasis, C57BL/6 mice had increased frequencies and numbers of airway macrophages in male compared to female mice [7]. On the other hand, female mice lungs had higher tissue‐resident CD103^+^ dendritic cells (DCs), both in terms of numbers and frequency, compared to male mice. Interestingly, CD103^+^ DCs are essential for cross‐presentation and for initiating lung anti‐viral responses by expanding effector T cells [7]. This may explain why males are at a greater risk of developing viral infections.

Plasmacytoid dendritic cells (pDCs) are key immune cells essential for blocking viral infection by producing high levels of type I interferons, downstream of signaling through toll‐like receptor 7 (TLR7), which is an X‐linked gene [8]. Accordingly, female pDCs produce higher IFN‐α compared to male pDCs, and in humans, pDCs are considered protective against COVID‐19‐associated pneumonia, a finding that may partially account for the higher prevalence and severity of the infection observed in males [9].

Furthermore, in naïve mice, group 2 innate lymphoid cell (ILC2) numbers, as well as CD4^+^ tissue‐resident memory cells, were greater in female lungs compared to males [7]. In response to IL‐33‐induced lung inflammation, an increased number of ILC2s and eosinophils were recruited in the lungs of female mice compared to male mice. This was accompanied by increased production of type 2 (T2) cytokines and chemokines [10]. During IL‐33‐driven acute lung inflammation, IL‐33 elicited a stronger mast cell response in female mice compared to males, and this sex‐associated difference was reversed in castrated mice [11]. Additionally, mast cells isolated from female mice demonstrated greater responsiveness to IL‐33 than those from male mice. These findings indicate that IL‐33‐mediated inflammation displays female‐dominant sexual dimorphism [11]. Furthermore, CD8^+^ tissue‐resident memory T cells (TRM), which reside in various barrier sites such as the lungs, are critical orchestrators of anti‐viral immunity [12] and the spatial tissue location of these cells governs TRM diversity and their function in the immune system [13]. A study by Poon et al. showed that in two different viral infection models, sex hormones regulated tissue‐resident CD8^+^ T cells differently [14]. Male donors maintained higher frequencies of flu‐specific CD8^+^ tissue effector memory (TEM) cells in the spleen and lung, whereas in CMV infection, males harbored higher frequencies of CMV‐specific TEM cells in the lung and lung‐draining lymph nodes (LLN) compared with females [14]. In contrast, flu‐specific CD8^+^ T Effector Memory Re‐expressing CD45RA (TEMRAs) in the spleen and lung, and CMV‐specific TEMRAs in the lung and LLN were higher in female donors compared to males. This suggested that viral infections contribute to virus‐driven T cell differentiation and maintenance differently in individual tissue sites [14]. Conversely, in a mouse model of hepatocellular carcinoma, anti‐tumoral CD8^+^ T cell responses were reduced in male mice, leading to increased tumour burden [15].

With respect to humoral immune responses, a human‐only ex vivo study demonstrated that B cell numbers in the human blood were similar between the sexes, but between puberty and menopause, females displayed increased numbers of class‐switched and memory B cells, which were hyper‐efficient at producing antibodies during puberty [16, 17]. This suggests that estrogen may play a regulatory role in B cell development [17].

Furthermore, in an ovalbumin (OVA) challenge mouse model, lung B cells mounted a higher OVA‐specific IgE and IgG1 response in female mice than in male mice [18]. Therefore, female mice are able to mount a more robust and elaborate antibody response compared to male mice.

Sex differences in immune cell composition may vary between tissue types and their function in an immune response is further shaped by their anatomical location. This is well demonstrated in a mouse model of adipose tissue inflammation, where Visceral adipose T regulatory cells (VAT) were enriched in male mice's adipose tissue but not in female mice's adipose tissue. Moreover, VAT Tregs were strikingly distinct in phenotype, transcriptional landscape, and chromatin accessibility compared to those in female mice [19]. Increased inflammation in the male VAT facilitated the recruitment of Treg cells via the CCL2‐CCR2 axis. Mechanistically, the authors found that androgen signaling regulated the differentiation of a unique subset of IL‐33‐producing stromal cells specific to the male VAT, which influenced Treg expansion in a B lymphocyte‐induced maturation protein 1 (Blimp1) transcription factor‐dependent manner [19].

Therefore, the literature suggests that different tissues may be exposed to unique environmental challenges and may therefore need to functionally adapt to meet those distinct tissue‐specific immunological demands.

### 1.3 Sex Differences in Lung Structural Cells (Figure 1)

The influence of biological sex on the function of lung structural cells is poorly investigated. Airway structural cells are key initiators of lung immune responses and the precise sexual dimorphism in their function remains undefined. Here, we highlight recent research investigating sex differences in immune responses by lung structural cells to external environmental stimuli (Figure 1).

The airway epithelium is an important structural barrier in the lungs, but also initiates immune responses to inhaled substances such as allergens, viruses, bacteria, and pollution particles. In this respect, transcriptional analysis performed on murine female versus male tracheal epithelial cells (mTECs) at steady state determined that females have higher expression of genes associated with increased barrier function and higher ciliary function compared with males [20]. Chronic exposure to cigarette smoke disrupted the epithelial barrier function in both males and females, while female mTECs exhibited unique transcriptional signatures of dedifferentiation with increased basal cells and markers of cellular senescence compared to males [20].

In addition, single‐cell RNA sequencing analysis revealed sexual dimorphism in human airway epithelial cells obtained from COPD patients. The authors found that smoking induced large transcriptomic changes in the airway epithelium between females and males, particularly the viral response and autophagy pathways [21]. Specifically, female smoker's epithelial cells harbored genes significantly enriched in autophagy and response to virus and type 1 interferon signaling pathways compared to male smokers [21].

Others have also demonstrated sex differences in the pulmonary microvascular endothelium. Human pulmonary microvascular endothelial cells (HPMECs) from healthy male and female donors, cultured under physiological shear stress, were analyzed using RNA sequencing and label‐free quantitative proteomics [22]. Gene expression showed sex‐specific pathways in both normoxic and hypoxic conditions, including those involved in cell proliferation. Female HPMECs exhibited a lower proliferation rate than male HPMECs [22]. Among these genes, thrombospondin‐1, an inhibitor of proliferation, was expressed at higher levels in female cells compared to male cells [22].

Lung fibroblasts are key initiators of pulmonary immune responses and, in the context of lung diseases such as asthma, are crucial for regulating type 2 lymphocyte expansion and function [23]. In a recent study by Stölting et al., estrogen was found to amplify type 2 airway responses in female mice compared to male mice following early‐life chronic exposure to house dust mite (HDM) [24]. E2 stimulated the production of interleukin‐33 (IL‐33) by lung fibroblasts and epithelial cells leading to the upregulation of epidermal growth factor receptor (EGFR) expression on T helper 2 (TH2) cells, resulting in enhanced IL‐5 and IL‐13 production [24].

In a neonatal mouse model of pulmonary hyperoxia, sex differences were observed in fibroblast proliferation and fibrotic gene expression. The gene expression data showed that male fibroblasts show greater activation of the Notch signaling pathway, which is considered to drive pro‐fibrotic responses [25].

Overall, this area of research is poorly investigated and leaves a critical gap in our understanding of how lung immune responses arising from immune‐stromal cell cross‐talk develop between the sexes in disease settings.

## 2 Multiple Layers of Genetic, Hormonal, and Environmental Factors that Impact Immunity (Figure 2)

**FIGURE 2:**
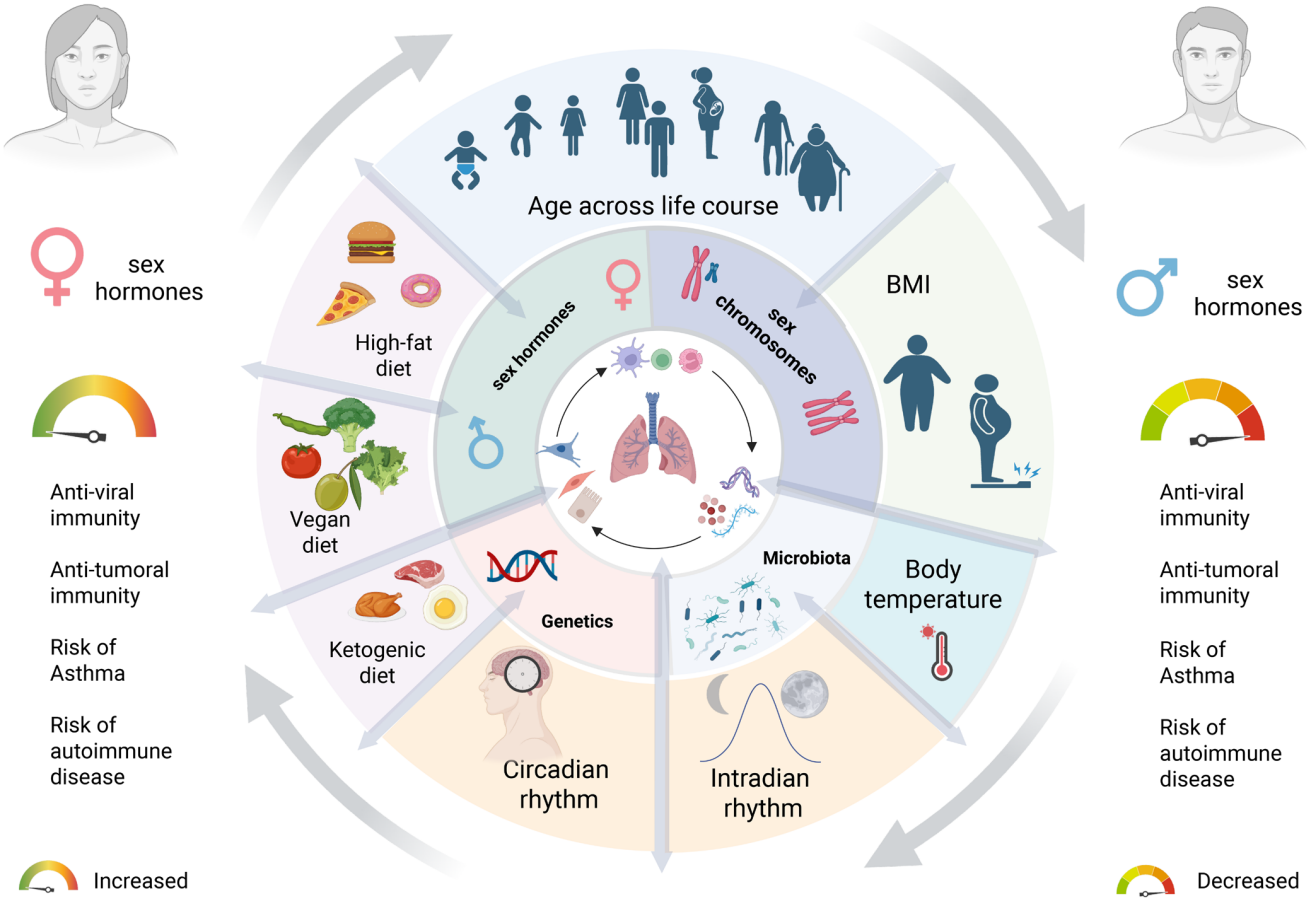
**Multiple Layers of Genetic, Hormonal, and Environmental Factors that Impact Immunity**. The magnitude and quality of immune responses across tissues are regulated by multiple interconnected factors. A key aspect of this immunoregulation is the interaction between cells and proteins. Sex hormones and the sex chromosomes serve as significant modulators of tissue immunity, with their effects further shaped by variables such as age, diet, genetics, circadian and infradian rhythms, body temperature and body mass index (BMI). These factors contribute to observed sex differences in tissue immunity. For instance, type‐I IFN responses tend to be higher in females than in males, leading to reduced rates of viral infections and cancers in females but also increasing susceptibility to inflammatory diseases, including asthma and autoimmune disorders, compared to males. Created with https://BioRender.com.

Sex differences in lung immunity are exquisitely regulated by a network of factors, including genetics, sex hormones, and environmental factors, which communicate to shape functional cell–cell interactions and cell–matrix interactions, lung development, homeostasis, and pathology (Figure 2). Therefore, lung immune cell function must be interpreted in its cellular and tissue context.

### 2.1 Genetics

Biological sex shapes the function of the immune system through differences in both immune and nonimmune cells, with these effects further influenced by tissue‐specific factors [26]. Sex is generally determined by genotype, with XX denoting female and XY denoting male. Less common genotypes, such as XXY, XYY, or XO, are associated with altered disease susceptibility [27]. Autoimmune disorders, including systemic lupus erythematosus, are correlated with increased X chromosome dosage, indicating a direct role for the X chromosome in regulating immune cells.

The X chromosome is approximately three times larger than the size of the Y chromosome and harbors approximately 800 protein‐coding genes, many of which are involved in immunity [16, 28]. To balance gene dosage, most genes on one X chromosome in females are silenced by inactivation. However, 23%–30% of inactive X (Xi) escapes inactivation, with some escaping only in certain tissues [29, 30]. For instance, *ZBED2* and *USP11* are tissue‐specific escape genes that regulate T cell exhaustion in tumors and control regulatory T cell function, respectively. In contrast, other genes always escape inactivation; these often encode proteins driving innate and adaptive immunity [31]. Additionally, some are in homologous pseudoautosomal regions of the X and Y chromosomes, where escape from X‐inactivation prevents haploinsufficiency [29]. As a result increased expression from escape benefits certain genes, for example *TLR7*, which recognizes viral single‐stranded RNA, is encoded only on the X chromosome and often escapes inactivation, resulting in a copy on each X chromosome in females [32].

This is particularly important in the context of lung viral infections such as SARS‐CoV2, where genome‐wide association studies have demonstrated that different CpG methylation patterns at the *TLR7* locus differ between females and males, rendering males more susceptible to infection [33].

### 2.2 Sex Hormones

Sex hormones, including estrogens, progesterones, and androgens, influence molecular changes in immune cells [34, 35]. Female and male sex hormone levels strongly fluctuate across the life course [34]. Moreover, in females, levels of sex hormones can change throughout the menstrual cycle and with the use of hormonal birth control or hormone replacement therapy [36]. These hormonal fluctuations contribute to the variability of sex‐based differences in immune function.

Others have reported that assisted reproductive technology (ART), in women, can significantly impact immune responses in the offspring. A study by Weinstock et al. showed that fertility treatment in women significantly increased infection‐associated morbidity in the conceived offspring compared to offspring delivered from mothers not on any fertility treatment [37].

Both peripheral and tissue resident immune and structural cells express estrogen receptors (ERα and ERβ), androgen receptors (ARs), and progesterone receptors (PRs), and respond to hormonal signaling. Previous reports have extensively documented how innate and adaptive immune cells express ER, AR, and PR signaling and respond to sex hormones [34]. Generally, estrogen enhances immune activity, whereas testosterone exerts immunosuppressive effects in both circulating and tissue‐infiltrating immune cell populations.

In addition to effects on the immune system there are direct associations between sex hormones and lung function. Using the UK Biobank, a positive association was found between testosterone and FEV1 and FVC in men, but not in women, although longitudinal analyses showed that women with higher levels of testosterone at baseline showed reduced decline in both FVC and FEV1 over the follow‐up period [38]. A more stringent analysis involving Mendelian randomization, which minimizes the observational effects, revealed that higher testosterone levels lead to higher FEV1 and FVC levels in both men and women from the general UK population [39]. In contrast, the sex hormone binding globulin (SHBG) has a negative effect on FEV1 and FEV1/FVC in men. These findings suggested that testosterone supplementation might be beneficial in people at risk of accelerated lung function decline, while screening for lung function deficits in those with lower testosterone levels may be prudent.

### 2.3 Environmental Factors

The lung immune defense is constantly encountered by host extrinsic environmental factors such as allergens, microbes, pollution particles, chemicals, etc. and the outcome of the immune response is strongly influenced by multiple other factors, including biological sex, age, microbiome status, diet, nutrition, circadian rhythm, and behavior across the human life course [40]. Notably, males and females exhibit distinct gut and lung microbiome compositions, and sex‐specific microbiome differences have been shown to influence the development of autoimmune disease in animal models [41].

Incorporating biological sex as a critical immunological variable in experimental design, analysis and interpretation is essential for both human and animal studies. Major international funding agencies mandate the inclusion of both sexes in all proposed experiments in grant applications. Additionally, designing human lung immunological studies with experiments conducted on transgender individuals undergoing sex hormone therapy may provide valuable insights into the effects of artificial hormone replacement therapy on lung immune function [40]. These studies can elucidate the dynamic relationships among sex chromosomes, sex hormone signaling, commensal microbes and alterations in immune pathways throughout the lifespan, while also considering environmental factors such as age, body mass index, diet and circadian and infradian rhythms (Figure 2).

#### 2.3.1 Sex Differences in the Lung and Gut Microbiome

Recent work by Chi et al. has found that tissue immunity is regulated by the integrated action of sex hormones and the microbiota, with sex hormones controlling the strength of immune responses and microbiota calibrating the tone of the immune response [42].

The microbiome is comprised of bacteria, viruses, and fungi impacts immune responses in multiple diseases, including lung disease and exerts an exquisite influence on immune development. Differences in lung structure, environmental exposures, diet, hormones, and medicinal supplements or biologics may alter both microbial composition and diversity within the tissue, thus modulating lung immune cell function. The interplay between sex hormones and microbes may impact bacterial growth, signaling, and metabolism [43]. The microbiome composition and diversity may differ over the lifespan and across phases with spikes in hormone levels, such as, the reproductive phase, puberty, pregnancy, and menopause.

In addition, the gut microbiome can influence lung immunity [44] and sex differences may exist in the gut microbial community structure and function.

In a mouse model of allergic airway inflammation and following dietary vitamin D supplementation of mice, differences in the lung microbiome were observed between male and female mice [45]. A study of patients with cystic fibrosis found that an increase in exacerbations coincided with the menstrual cycle and was associated with higher estradiol levels. Conversely, women taking oral contraceptives had fewer exacerbations [46].

A separate study investigated the effects of aztreonam, a beta‐lactam antibiotic targeted against gram‐negative bacteria, including *Pseudomonas aeruginosa* (PA), a prevalent lung microbiome species associated with cystic fibrosis (CF) [47]. The authors reported that males exhibited a significantly higher Shannon diversity index, indicating a more diverse microbiome, which correlated with a lower abundance of PA and increased composition of Stenotrophomonas, Streptococcus, Dialister, and Shuttleworthia. In contrast, females showed higher Pseudomonas abundance and a greater tendency to respond to aztreonam [47].

Another study analyzed the lung microbiome in patients with lung adenocarcinoma (LUAD) versus lung squamous cell carcinoma (LUSC) [48]. The results indicated that tumors harbored unique, significantly dysregulated microbial populations based on both lung cancer type and the biological sex of the patient. In young males with LUSC, *Pseudomonas putida* was the most prominent bacterial species. In contrast, all female LUAD patients exhibited distinct bacterial species not observed in LUAD males [48].

These findings uniquely imply that bacterial species may serve as accurate diagnostic biomarkers for specific lung cancer types, as their presence appears to be influenced by patient sex.

In addition, because of the lungs anatomical location, the pulmonary microbiome is strongly influenced by microbial communities in the oropharynx and gut, and the interactions among oral, gut, and lung microbes can strongly shape the outcome of lung immune responses.

In a mouse model of HDM‐induced allergic airway inflammation investigating the role of the lung‐gut axis in allergic responses, intranasal administration of allergen induced sex‐specific changes in the microbiome of the gut and lung and induced distinct differences in lung pathology between the sexes. Females showed significantly higher inflammation and histopathological changes compared to males after HDM exposure, but males presented with higher airway hyperresponsiveness (AHR) than females [49]. Furthermore, analysis of fecal samples revealed that alterations in the gut microbiome influenced sex‐specific changes in lung gene expression and associated pathways. These gut microbiome alterations were associated with the *Firmicutes*‐to‐*Bacteroidetes* ratio (F:B), which was significantly lower in male mice than in females after HDM challenge. Furthermore, the microbial alpha diversity was increased in males but decreased in females after HDM exposure [49].

Overall, the literature suggests that sex hormones may influence lung immunity and this relationship is intertwined with alterations in the gut microbiome and the gut‐lung axis.

#### 2.3.2 Dietary Contributions to Sex Differences

Diet plays a critical role in regulating sex‐specific effects on immune responses and maternal diet can have long‐lasting effects on offspring's lung health. In the Western world, diets often incorporate foods that are high in fat, lower in fiber, with a high‐salt content. Studies in mice showed that an excessive maternal high‐salt diet led to increased susceptibility to lung fibrosis in male offspring, whereas female offspring showed distinct changes in metabolic and immune pathways [50]. These findings highlight that early‐life is a critical developmental window when nutrition programs sex‐specific vulnerabilities to lung disease.

Furthermore, the amount and type of fiber consumed can influence pulmonary responses through the gut microbiome. Previous work by Trompette et al. showed that groups of mice fed a diet with varying fibre levels and exposed to HDM had distinct immune outcomes in allergic airways disease. The authors observed that lung inflammation was lower in mice consuming high levels of fermentable fiber than in those on a low‐fiber diet. Interestingly, the animals also harbored a community of intestinal microbes that generated higher levels of short‐chain fatty acids when metabolizing fiber. These fatty‐acid molecules boosted the generation of dendritic cells that were less able to trigger allergic inflammation in the lungs [51]. In this respect, how gut‐derived metabolites specifically modulate lung immune responses remains unclear and will be an essential area of investigation moving forward.

Similarly, Tashiro et al. showed that after ozone‐induced airway hyperresponsiveness, the gut microbiome contributed to sex differences in ozone‐induced airway hyperresponsiveness. Male and female mice were fed on a diet free of fiber or diets enriched with either cellulose or pectin for 3 days prior to ozone exposure. Cellulose‐enriched diets reduced ozone‐induced airway hyperresponsiveness in male mice compared to control or pectin‐enriched diets, but not in females. Conversely, fiber‐free diets increased ozone responses in females, but not in male mice [52]. These differences were attributed to sexual dimorphism in the impact of dietary fiber on the gut microbiome and identified specific bacterial species associated with ozone‐induced airway hyperresponsiveness. Therefore, diet can differentially modulate the actions of sex hormones in shaping lung immunity and is an important consideration for respiratory health. Recent trends in dietary habits including vegetarian, vegan, high‐fat and ketogenic diets have profound influences on immune responses. Indeed vegan diets were associated with antiviral immune responses whereas ketogenic diets contributed to alterations in adaptive immune responses [53]. In this respect, our understanding of how current dietary trends influence lung immunity remains unclear and warrants further interrogation.

#### 2.3.3 Sex Differences in Circadian Rhythm

Circadian rhythms are internal physiological variations driven by feedback loops that regulate gene expression in 24 h cycles [54]. The suprachiasmatic nucleus in the hypothalamus acts as the master pacemaker, receiving light and dark cues, aligning the system with the solar day, and transmitting timing signals throughout the body via autonomic and endocrine pathways. In addition, the sympathetic nervous system synchronizes circadian rhythms in cells and tissues by coordinating tissue‐specific oscillations in immune cell recruitment. These rhythms originate and persist at the molecular, cellular, tissue, and organ levels, via received circadian cues from feeding and other behavior [55]. A well‐synchronized circadian system allows the body to anticipate and manage regular physiological demands.

Sex differences in the circadian system may contribute to varying susceptibility and disease risk between men and women. Exposure to artificial light, irregular sleep patterns, time zone travel, shift work, and other contemporary factors can disrupt circadian rhythms. Such desynchronization leads to short‐term impairments in physiological functioning and, over time, increases the risk of diabetes, cardiovascular disease, and cancer, particularly among women [56].

Circadian rhythms and sex differences contribute to the pathophysiology of asthma‐related lung inflammation. A recent study by Srinivasan et al. showed that, following chronic HDM exposure, differential effects on asthma pathology were observed between females and males depending on the time of the day (zeitgeber time: ZT0/6:00 a.m. vs. ZT12/6:00 p.m.) when mice were exposed to the allergen [57]. Exposure to HDM at ZT12 resulted in greater infiltration of eosinophil subtypes and elevated levels of related chemokines in female mice compared to males. Additionally, T_H_2 gene expression, cytokine release, and humoral immune responses were higher in females at ZT12 [57]. These findings indicate that airway inflammation is influenced by both time of day and sex‐based differences in HDM‐induced asthmatic phenotypes, including inflammation and remodeling. Circadian clock disruption may further worsen asthma outcomes in females compared to males.

Furthermore, a clinical study found that women experienced greater cognitive impairment than men experiencing disrupted sleep–wake cycles, suggesting that the ramifications are far greater than in men [56]. In line with this, fluctuations in the normal circadian rhythm may detrimentally affect females more than males.

In addition, sex hormones and clock genes have a bidirectional relationship with the circadian cycle controlling sex hormone synthesis and fluctuating with the time of day and the specific phase of the estrous cycle [58]. Sex hormones interact directly with the circadian system to regulate gene expression, influence biological processes, and modulate their own synthesis [58]. Thus, sex hormones and the circadian clock exhibit a bidirectional relationship and may differently impact lung immunity in females compared to males.

#### 2.3.4 Sex Differences in the Extracellular Matrix

The Extracellular Matrix (ECM) plays a pivotal role in regulating cellular behavior, tissue organization, and disease progression. Therefore, cell function cannot be understood in isolation from the surrounding tissue ECM microenvironment. Changes to the ECM are associated with most lung diseases, and most studies highlight that dysregulation of the ECM not only disrupts tissue integrity but also profoundly affects immune cell infiltration and localization [59].

The ECM is often overlooked in studies of tissue immunity because its components are insoluble, highly cross‐linked, and more challenging to extract and analyze.

Puttur et al. have previously shown that the lung ECM is crucial for regulating airway remodelling in early‐life chronic allergen‐induced lung inflammation [60] and for regulating immune cell behavior and function in acute lung inflammation [61]. Whether differences exist between the sexes in how the ECM develops, which ECM subtypes contribute to immune cell function and in tissue remodeling remains unclear.

A recent lung tissue research consortium profiled lung tissue gene expression and DNA methylation data from 747 individuals with COPD or healthy controls and identified sex‐ and disease‐specific differences in COPD‐associated gene regulation using gene regulatory networks. The authors found that genes involved in the ECM exhibit greater transcriptional factor targeting in healthy female subjects than in male subjects [62]. However, this pattern is reversed in COPD, with men showing stronger regulatory targeting of ECM‐related genes than women. Smoking exposure, age, lung function, and emphysema were all associated with sex‐specific differential methylation of ECM‐related genes [62].

Another study investigating the pathology of aortic valve stenosis (AVS) found that males more often develop valve calcification than females do who more often develop valve fibrosis [63]. Interestingly, the authors found that the Y chromosome modulates male‐specific valvular interstitial cells (VICs) to aberrantly activate myofibroblasts during AVS, driving the fibrotic valve phenotype in females. Conversely, in males, myofibroblasts further differentiate into osteoblast‐like cells and produce calcium nanoparticles, thereby driving valve calcification in males [63]. The authors found that male VICs responded to nanoscale ECM cues to promote an osteoblast‐like cell phenotype, suggesting that sex chromosome‐linked genes can drive disease progression by responding to nanoscale ECM cues.

With regards to ECM regulation of immune function, gene expression changes in the ECM do not always translate to protein production. Hence, with the advent of bespoke sequential extraction workflows that enable comprehensive analysis of both cellular and extracellular protein compartments [64] and the identification of sex‐specific differences, proteomic maps can be generated to guide selection of cell–matrix interactions between sexes. Furthermore, to preserve spatial information, live tissues could be obtained after imaging for multiplexed proteomic analysis. Tandem Mass Tag (TMT) 18‐plex labeling [65] of lung slices would allow for simultaneous quantification across multiple lung regions, providing spatially resolved ECM profiles that reveal regional molecular differences between the sexes. This integrative approach will enable direct correlation between spatial proteomic profiles and high resolution microscopy of cell‐matrix patterns, advancing our understanding of how local matrix environments guide immune cell responses in diseased lung tissue between sexes. Therefore, environmental factors play a critical role in controlling the interaction between sex hormones and the immune system with strong impact on outcomes of lung disease and infection.

In addition to the above environmental factors influencing the interactions between sex hormones and lung immunity, body temperature may be another environmental factor warranting attention. In recent years, body temperature has been found to be sexually dimorphic. The body temperature of females is 0.1‐0.5°C warmer on average than males [66] and regulates tissue immunity through activation of highly conserved fever and heat shock pathways affecting the metabolism of immune cells [66]. In this respect, how differences in body temperature between the sexes impacts lung immunity remains poorly understood. With the challenges of global warming and the impact of climate change, it is essential to interrogate how body temperature modulates lung and systemic immune responses and how sex hormones contribute to these interactions between females and males.

## 3 Sex Differences in Development of Respiratory Disease Outcomes (Figure 3)

**FIGURE 3:**
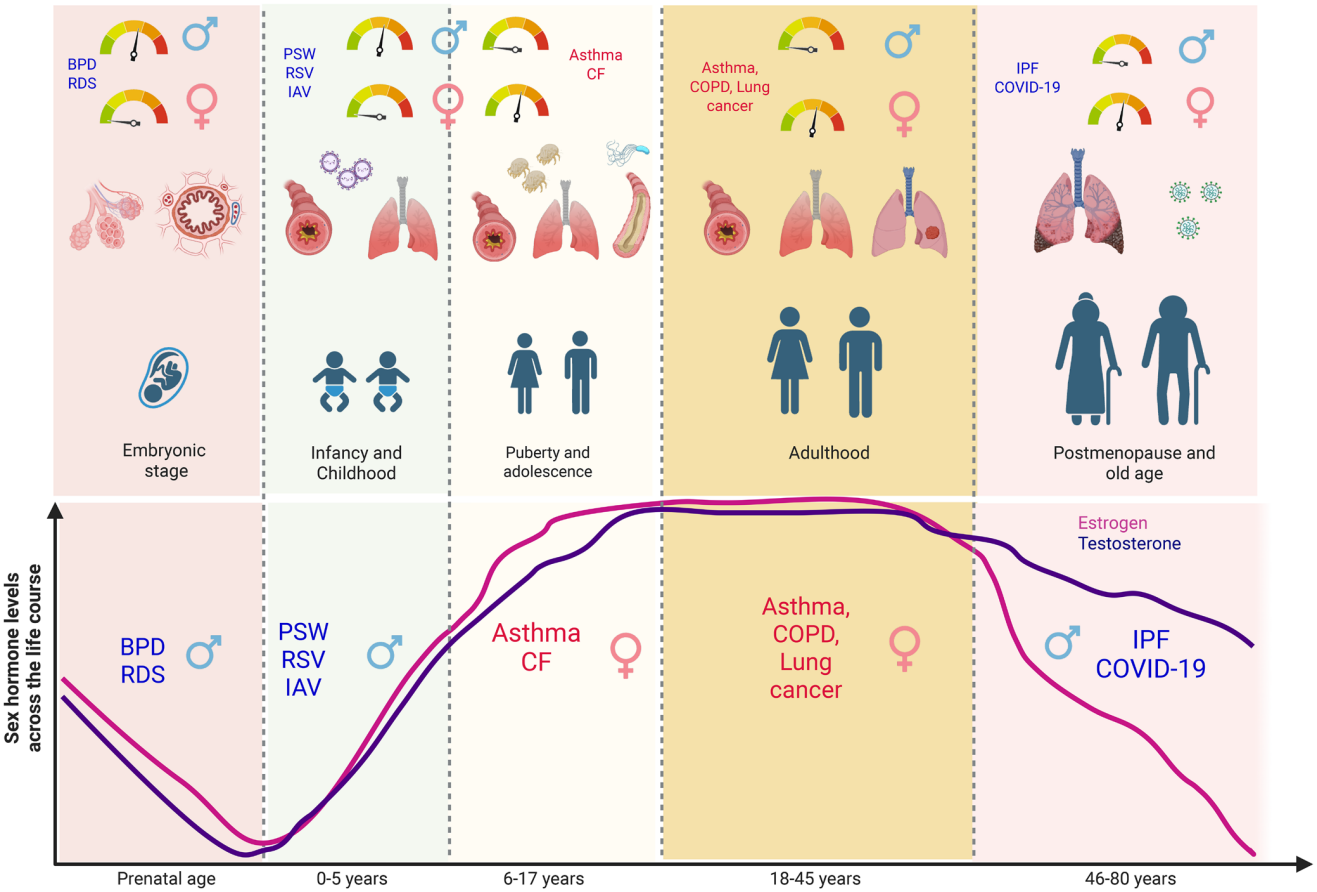
**Interplay between sex hormones, age, infection risk and development of lung disease.** Sex hormones such as estrogen (magenta) and testosterone (blue) play a vital role in lung development and function across the life course, from prenatal lung maturation through to postnatal early life, puberty, adulthood and old age. The fluctuations in these hormone levels in the body can alter the outcome of lung disease susceptibility in females (magenta) and males (blue) and this is well demonstrated in lung disease such as asthma, where at prepubertal age males are more susceptible to developing the disease, while post puberty this reverses and women are more susceptible to developing asthma. This is primarily because of a spike in estrogen levels, which are known to drive type 2 immune responses in females. The susceptibility to and development of lung disease and lung infection are strongly regulated by an individual's sex hormone status and age. Created with https://BioRender.com.

Sexual dimorphism in lung immunity to respiratory disease arises from dynamic changes in early lung development that shape lung structure and composition. From the 16th week of gestation through to adulthood, males and females show distinct lung immune responses [67]. These differences are largely driven by variation in sex hormone levels during developmental changes across the life course including puberty, pregnancy and menopause, which also influence lung function and health [67]. In the first weeks of life, female sex hormones promote lung development and maturation, whereas androgens impede lung development [68]. After puberty, this pattern reverses in conditions like severe asthma, where higher androgen levels are protective and variation in female sex hormones during the menstrual cycle can worsen asthma [67]. Current research shows that the effects of sex hormones on lung health depend on the timing of exposure, leading to sex‐specific differences in disease prevalence and severity throughout life. In this section of the review, we summarize the literature on sexual dimorphism in respiratory diseases and infections.

### 3.1 Sex Differences in Development of Chronic Respiratory Diseases (Figure 3)

#### 3.1.1 Preschool Wheeze

Allergic airway diseases are common in early‐life, with approximately 10% of children presenting with recurrent wheeze [69]. About half of all children develop wheezing upon viral infection in the first 5 years of life, but only one‐third of those will go on to develop asthma [70]. Understanding the environmental triggers that drive the transition from preschool wheeze (PSW) to school‐age asthma (SA) is an important clinical question that remains unanswered. A population‐based cohort study carried out on preschool wheeze children showed that prematurity, maternal severe asthma, and atopy were high‐risk factors for developing childhood asthma [71]. However, sex hormones play a crucial role in exacerbations of wheeze and asthma‐associated symptoms. Boys showed a higher frequency of developing viral‐induced wheeze‐like symptoms (median age of 3–5 years), but this male predominance decreased with age [72]. The sex‐associated changes in wheeze development are associated with differences in the development of cytokine responses [73]. Among children who experienced wheezing during their third year, boys exhibited elevated IFN‐gamma, IL‐5, and IL‐13 responses at age three [73]. These elevated cytokines in boys were associated with increased rates of sensitization, total IgE levels, and peripheral eosinophil counts [74].

Thus, biological sex is an important modifier of disease risk for childhood wheeze and wheezing phenotypes differ between males and females in pre‐pubertal children [75].

#### 3.1.2 Respiratory Distress Syndrome

Respiratory distress syndrome (RDS) occurs in premature infants as a result of pulmonary surfactant deficiency [76]. Affected infants typically exhibit widespread alveolar atelectasis and decreased lung compliance, which are frequently accompanied by complications such as pneumothorax. Before the introduction of antenatal corticosteroids and postnatal surfactant therapy, RDS resulted in a substantial increase in neonatal deaths, particularly among male newborns [77].

The roots of RDS lie in surfactant deficiency and therefore immature lung development, both of which are more common in male infants of the same gestational age [76]. With less developed lungs, these newborns are at a greater risk of disease after birth. Notably, pulmonary surfactant appears earlier in females, since estrogen promotes the production and release of surfactant while male hormones impede surfactant production. These differences underscored by a comprehensive meta‐analysis of 685 preterm infants which found thatin a cohort of preterm infants who had succumbed to RDS, males dominated the group, with a mortality rate 8.5 times higher than in females [78]. The same study found that boys also face a higher risk of developing lower respiratory tract infections including bronchiolitis, and pneumonia. To prevent and treat RDS, clinicians employ postnatal surfactant and antenatal corticosteroid therapy.

#### 3.1.3 Bronchopulmonary Dysplasia

Bronchopulmonary dysplasia is a lung disease commonly associated with premature infants. BPD is identified by aberrant pulmonary vascular development and arrested alveolarization [79, 80]. Premature neonates are diagnosed at 36 weeks of gestational age by assessing their need for assisted mechanical ventilation and for oxygen support [81]. The incidence rate is common in neonates with low birth weight [82].

Thanks to advances such as antenatal corticosteroids, exogenous surfactant, and gentler ventilation, more extremely preterm infants have an improved rate of survival. Yet, this progress has come with a price: the incidence of BPD has climbed over recent decades. While fewer children now die from the disease, those who survive often face a lifetime of lung challenges, from tracheostomies and ongoing ventilation to pulmonary hypertension and developmental hurdles. As these children grow, studies reveal that adults born preterm continue to struggle with airflow limitations, gas trapping, and poor gas exchange compared to their peers born at term. The decline in lung function often continues through childhood, especially in males who present with higher disease‐associated mortality, greater oxygen dependency, increased pulmonary hemorrhage, and more frequent use of steroids in the postnatal period compared to females [79, 82, 83]. The underlying mechanisms driving the sexual dimorphism in susceptibility to BPD remains poorly understood. Previous research has shown that dysregulation in pulmonary angiogenesis may contribute to greater male susceptibility to BPD [84]. Others have demonstrated that in a mouse model of BPD, microRNA‐30a may be an essential factor in these observed sex‐specific differences in susceptibility to BPD [85].

#### 3.1.4 Asthma

Asthma is a chronic airways disease characterised by airway inflammation and irreversible airway remodeling. Symptoms include cough, shortness of breath, wheezing, chest tightness, and airflow limitation [86]. Asthma casts a significant burden on the individual experiencing the symptoms, diminishing their quality of life and contributing to disease‐associated mortality in both women and men [87]. Across the lifespan, the risk, prevalence, and severity of asthma vary dramatically between the sexes. In childhood, boys are more likely to develop asthma than girls, while after puberty, females are at a greater risk.

In females with asthma, strong evidence links disease severity to hormonal changes during puberty, menstrual cycles, pregnancy, or menopause. In a recent study adopting a neonatal mouse model of early‐life allergic airway inflammation, Stölting et al. showed that following sexual maturity, female mice exhibited increased pulmonary type 2 (T2) immune responses and higher IL‐33 levels than males [24]. Mechanistically, this was attributed to epidermal growth factor receptor (EGFR) signaling. EGFR signaling was essential for T2 cytokine production by CD4^+^ T cells, and in vivo EGFR inhibition with gefitinib significantly reduced female T2 responses, eliminating sex differences [24]. Furthermore, treating male mice with estrogen also increased T2 responses to HDM, reducing differences between sexes [24]. These data were the first to provide a link between estrogen and EGFR signaling in promoting T2 immunity in allergic inflammation. Previous studies by Cephus et al. showed that estrogen is important for driving T2 immunity by binding to the ERα receptor on ILC2, driving ILC2‐mediated airway inflammation. The Cephus study showed that after exposure to acute fungal allergen, estrogen increased IL‐33 expression in bronchial epithelial cells [88]. Stoelting et al. showed that E2 also stimulates IL‐33 production in airway fibroblasts, which increases EGFR and ST2 expression on T_H_2 cells and that EGFR signaling is required for cytokine production by these cells. Stölting et al. observed increased intracellular ST2 after IL‐33 stimulation, which increased ST2 expression in a larger proportion of CD4^+^ T cells [24]. Expression of EGFR on activated CD4^+^ T cells facilitated the production of IL‐13 in response to IL‐33 stimulation and was mediated by the direct interaction between EGFR and ST2 [24]. This response was similar in CD4^+^ T cells stimulated with HDM or IL‐33, suggesting that EGFR signalling may promote antigen‐specific T2 cytokine production and suggesting that T_H_2 cells are dependent on EGFR signaling [24]. The Stölting study showed for the first time that following allergen exposure, sex hormones, in particular estrogen, induces IL‐33 from lung fibroblasts and bronchial epithelial cells, which in turn increases EGFR‐mediated regulation of T2 cytokines in CD4^+^ T cells and may explain the severity of asthma symptoms in females than in males [24].

T2 responses have also been observed in clinical studies involving severe asthma. Previous reports have demonstrated that patients with asthma show increased numbers of ILC2s in their blood and airways [89, 90, 91, 92]. Moreover, a recent study by Mincham et al. used a comprehensive panel of ILC‐defining markers, combined with high‐parameter flow cytometry to unbiasedly investigate the phenotypes and functional diversity of ILCs in healthy individuals and patients with severe asthma (SA). In addition, the authors investigated the relationship between ILCs in peripheral blood versus airway compartments [93]. Mincham et al. showed that unsupervised analysis of ILC clusters that were stratified by sex identified IL‐13^+^ ILC2s, which were increased in females with severe asthma compared with their male counterparts. Interestingly, the authors found that these differences persisted even in patients receiving anti–IL‐5/5Rα biologics. Murine studies have previously shown that androgens negatively regulate ILCPs and underlie heightened ILC2 responses in female animals [88, 94]. Consistent with these findings in humans, Mincham et al. demonstrated that total blood ILCPs in SA patients inversely correlated with plasma testosterone irrespective of disease state [93]. Similarly, the frequency of ILC2s was found to be lower in male asthmatics, and functional expression of androgen receptor on ILC2 of both sexes suggests that they are programmed to respond to androgens [95]. Interestingly, androgen insensitivity syndrome is strongly associated with enhanced asthma risk [96].

Loewenthal et al., compared cohorts across two independent data sources spanning UK primary and secondary care and found that women with asthma had increased symptoms and were more likely to be obese despite higher FEV1% predicted and lower type 2 biomarkers with no significant difference in blood eosinophils or biological therapy [87]. These were consistent differences across both SA and mild/moderate asthma cohort, suggesting that many of the sex differences seen in SA also exist in patients with mild/moderate asthma [87].

Overall, these studies reveal that women are at greater risk of developing asthma after puberty and have increased in risk of worsening lung health compared to men. Clinical studies and mouse models report that estrogen can activate lung inflammation and allergic reactions, while androgens can reduce inflammation [97, 98]. Asthma severity in men increases in older age as androgen levels decline [99].

#### 3.1.5 Chronic Obstructive Pulmonary Disease

Chronic Obstructive Pulmonary Disease (COPD) is a chronic respiratory condition characterised by cough, airway remodeling, and lung parenchymal destruction [100]. COPD is a global problem affecting approximately 174 million individuals worldwide, including 104.7 million men and 69.7 million women [67]. The World Health Organization projects that COPD will become the third leading cause of death globally by 2030. While COPD prevalence is rising in both sexes [101]. Increased tobacco usage is a strong precursor for COPD development. Although originally COPD was associated with men, recent years have seen an increase in prevalence among women due to increased tobacco use by both sexes [102].

The importance of sex differences in COPD disease presentation has been increasingly recognized in recent years. Studies indicate that women with COPD experience greater disease burdens as well as different disease‐associated symptoms and pathology compared to men. Younger women develop COPD more often and have more frequent respiratory exacerbations [103] and since 2000, COPD‐related mortality has been higher among women than men [104].

Multiple social and environmental factors have contributed to the increasing ubiquity of COPD among women. In addition to increased tobacco use, longer life expectancy, and changing patterns of occupational exposure have played significant roles [102]. Furthermore, indoor air pollution from accumulated biomass fuel during cooking poses a significant risk and may contribute to COPD in neversmokers, with women increasingly affected [105].

The immunological pathways underlying sex differences in COPD are complex. In mouse models of COPD, females develop more small airways disease, while males develop more emphysema when exposed to the same level of cigarette smoke [106]. In female mice, cigarette smoke exposure has been shown to activate transforming growth factor‐β (TGF‐β) and reduce the gene expression of antioxidants and the nuclear factor erythroid 2‐related factor 2 (Nrf2)–antioxidant response element (ARE) [107]. Dysregulation of the Nrf2‐ARE pathway is associated with the development of COPD [108]. Furthermore, in humans, COPD is associated with increased production of tumor necrosis factor alpha (TNF‐α), interleukin‐6 (IL‐6), C‐reactive protein, matrix metalloproteinase 9 (MMP‐9), pulmonary and activation‐regulated chemokine (PARC), and vascular endothelial growth factor (VEGF), which collectively are thought to drive COPD pathology [109]. Precisely how these immunological networks differ between the sexes remains unclear, and mechanistically how immune responses are regulated by sex hormones in COPD between the sexes needs further investigation.

#### 3.1.6 Cystic Fibrosis

Cystic fibrosis (CF) is a multiorgan disease caused by changes in the Cystic Fibrosis Transmembrane Conducatnce regulator (CFTR) gene. Research shows that sex differences exist in the way CF affects males and females differently, with females having more lung complications and a shorter life expectancy than males [110]. Precisely how sex contributes to different disease outcomes in CF between females and males remains unclear; however, multiple factors have been shown to contribute to the sex‐associated disease etiology. Factors contributing to sex‐based disparities in CF outcomes include diagnostic bias, anatomical differences, socioenvironmental influences, medication adherence, physical activity levels, and the effects of sex hormones [67]. Higher rates of illness and death from CF in females have been connected to less understanding of the disease outlook, higher anxiety, more psychological withdrawal, lower treatment adherence and less physical activity after puberty [111].

In CF, age plays an important role in the sex differences in the outcome of CF. This is reflected in females having a higher bacterial burden of *Pseudomonas aeruginosa* and antibiotic‐resistant *Staphylococcus aureus* compared to males from a young age [112]. Females have a higher likelihood than males of acquiring methicillin‐sensitive strains of bacteria, including Aspergillus species, *Staphylococcus aureus* , *Haemophilus influenzae* , and nontuberculous mycobacteria at a younger age, especially prior to puberty [113].

Maselli et al. showed that during puberty and the reproductive years, females present with higher rates of lung infection and experience more exacerbations, more pulmonary complications, and lower lung function [112, 114]. The mechanisms underlying sexual dimorphism in CF and how sex hormones regulate immune responses and disease outcomes remain poorly characterized.

Female sex hormones play an essential role in CF pathogenesis. Poor mucociliary clearance is characteristic of CF patient bronchial epithelial cells (BECs) due to dysfunction in the CFTR gene. Studies by Casavola et al. showed that BECs expressing F508del mutated protein when treated with Estrogen showed reversal of the functional defects of CFTR gene in CF BECs [115]. Conversely, the engagement of 17B‐estradiol with estrogen receptor increased MUC5B production from mucosal glands, contributing to mucous plugging in CF airways [116]. Additionally, 17β‐estradiol induced the conversion of *Pseudomonas aeruginosa* from a non‐mucoid to mucoid phenotype in females suffering from CF. Interestingly, overproduction of 17β‐estradiol in women resulted in the presence of higher mucoid strains of *Pseudomonas aeruginosa*, triggering enhanced pulmonary exacerbations [46]. The increase in post‐pubertal exacerbations can reflect stages of their menstrual cycle, with the luteal phase associated with higher lung function than other cycle phases [117]. Chotirmall et al. also showed that 17β‐estradiol treatment decreased the expression of proinflammatory cytokines via increased production of leucoprotease inhibitor (SLPI), which was associated with higher rates of infection in women compared to men [46]. In mice, 17β‐estradiol enhances toll‐like receptor 2, IL‐17A, and IL‐23 expression and elevates lung immune cell infiltration and lung mucin production [118]. Studies by Abid et al. showed that when *Pseudomonas aeruginosa* was administered to mice, female mice showed poor clearance of bacteria compared to male mice [119]. However, administering the estrogen receptor antagonist ICI‐182,780 accompanied by performing an ovariectomy, rescued the poor bacterial clearance in female mice [119].

In this respect, a limited number of studies have characterized the specific role of sex hormones on CF disease progression. Further research would be warranted to provide insights into how sex hormones regulate immune mechanisms in CF disease and regulate poorer survival in females.

#### 3.1.7 Idiopathic Pulmonary Fibrosis

Idiopathic pulmonary fibrosis (IPF) is a chronic, progressive fibrotic lung disease characterised by interstitial pneumonia, primarily affecting the elderly and is associated with scar tissue formation in the lungs. Current therapeutic measures include the use of two anti‐fibrotic therapies, pirfenidone and nintedanib [120]. However, following diagnosis, the median survival rate is 3–5 years, and most individuals experience progressive decline in lung function, dependence on assisted oxygen support, and reduced quality of life [121]. IPF has a greater prevalence in men, while the female sex is associated with improved survival outcomes [122]. Although the precise mechanisms governing IPF pathogenesis remain incompletely characterized, important cellular changes in the lung have been associated with the disease.

Epithelial cell dysfunction and abnormal epithelial‐mesenchymal signaling have been associated with IPF disease, leading to activation of fibroblasts, aberrant extracellular matrix deposition and tissue remodeling [123]. More specifically, IPF is accompanied by increased TGF‐β production, epidermal growth factor, and platelet‐derived growth factor primarily by alveolar epithelial cells in response to lung injury. These mediators play an essential role in differentiating fibroblasts into myofibroblasts, which synthesize and lay down aberrant ECM proteoglycans, driving lung fibrosis and tissue remodeling in the affected lungs [124, 125, 126].

Although these studies provide important insights into how lung cells and mediators collaborate to promote disease pathogenesis, the precise molecular mechanisms underlying sex‐specific regulation of these processes remain unclear. Kovacs et al. demonstrated that estradiol impacted the expression of genes associated with chromatin remodeling pathways [127]. Furthermore, genome‐wide association studies have identified single‐nucleotide polymorphisms in the A‐kinase anchoring protein 13, mucin 5B, and desmoplakin genes, which are genetic factors mediating sex differences in IPF [128].

With respect to sex hormones, studies in bleomycin injury models suggest that bleomycin induces an increase in the expression of different pro‐fibrotic factors and in male mice, it also increases concentrations of ERα (estrogen receptor α) while decreasing concentrations of ERβ in male mice, paralleling concentrations of these receptors in lung tissues of patients with idiopathic pulmonary fibrosis (IPF) [129]. Furthermore, Elliot et al. have shown that insulin‐like growth factor I (IGF‐1) and epidermal growth factor (EGF) can increase ERα independent of E2, suggesting that mechanisms independent of ligand‐dependent regulation of the estrogen receptors may promote IPF [129].

A clinical study evaluated the desaturation rate during a 6‐min walk test and overall survival between females and males with IPF. The study found that males with IPF experienced a more rapid decline in exertional desaturation over time than females. Furthermore, males had lower survival rates compared to females, and the survival advantage in females was apparent even after adjusting for changes in the exertional rate of desaturation and FVC [122].

The research to date highlights the crucial need for future research to incorporate a sex‐specific approach for diagnosing and managing IPF between males and females. In this respect, further exploration of the involvement of sex hormones in immune pathways controlling IPF pathogenesis will have important implications in IPF management.

#### 3.1.8 Lung Cancer

Lung cancer is a major global public health concern and is the second most common malignancy leading to mortality in women and men worldwide. Due to improved low‐dose computed tomography (LDCT) screening methods and therapeutic strategies, the rate of survival in affected cancer patients have improved. Between 2006 and 2015, the overall cancer mortality rates decreased by approximately 1.5% annually in both sexes [130]. Among lung cancers, the predominant types of cancers include either small cell lung cancer (SCLC) and non‐small cell lung cancer (NSCLC) [131]. The development of lung cancer subtypes is controlled by multiple environmental risk factors.

Among these risk factors, tobacco use [132], air pollution [133] and occupational carcinogens [134] have been implicated in lung cancer development. However, 85% of the cancers occur as a result of tobacco smoking [132]. Women are increasingly susceptible to developing lung cancer than men, even at different levels of cigarette smoke exposure, and females show higher polycyclic aromatic hydrocarbon‐derived DNA adducts than male smokers [135]. Although the literature suggests that females are at a greater risk of developing lung cancer, the biological drivers of this difference remain poorly defined. To further understand these differences, it is important to examine the underlying mechanisms.

Sex hormones play a vital role in the regulation of lung cancer. In particular, Estradiol (E2) has been shown to promote lung cancer [136, 137]. ERα and Erβ are receptors for estradiol, and Erβ is expressed on cancer cells in addition to the lung epithelium and fibroblasts. In contrast, ERα is poorly expressed in the lung, but in lung cancer, ERα expression is increased by epigenetic regulation and CpG methylation [138]. Similarly, lung cancer cell lines also show evidence of CpG methylation compared to normal cells [139], highlighting the importance of estrogen‐driven epigenetic regulation in lung cancer.

Other proposed mechanisms include cigarette smoke, which acts on the enzyme CYP1B1 in lung tissue, induces estrogen into reactive oxygen species that damage DNA and increase the risk of cancer when combined with tobacco compounds [140].

To delineate the immunological differences between sexes in lung cancer, a recent study performed a meta‐analysis of available lung cancer transcriptional datasets and found that genes associated with acute inflammatory response were enriched in women compared to men [141]. Furthermore, genes encoding for TLR7, FoxP3, and IL‐2 receptor were all enriched in female lung cancer and were all the genes associated with the X chromosome. This suggested that females showed a heightened immune activity and that cancer cells may thereby evade immune recognition [142].

Other studies performing single‐cell analysis found that tumor‐associated macrophages (TAMs) displayed greater antigen‐presentation capacity and elevated IFNγ levels resulting in higher antitumor activity in tumor cells from female patients [143].

Another study reported that genes associated with T‐cell dysfunction, inhibitory immune checkpoint molecules, and immune‐suppressive cells such, as Tregs and MDSCs, were more highly expressed in NSCLC tumors in women than in men [144].

Furthermore, smoking can profoundly affect the immune system. A study by Zhao et al. found that cigarette smoke can induce Mitogen‐Activated Protein Kinase (MAPK) signalling, which can affect the activation of the Nuclear Factor kappa‐light‐chain‐enhancer of activated B cells (NFkB) signalling pathway activation [145]. Additionally, smoking can reduce overall T cell activity [146]. Although the above studies highlight the importance of cigarette smoke in immune modulation, further studies are needed to precisely dissect the mechanisms underlying the effects of cigarette smoke on immune cell populations and immune function and may provide vital information into how immune responses are regulated during lung cancer development and progression between the sexes.

Given the link between sex hormones and lung cancer, hormone replacement therapy (HRT) is often prescribed for postmenopausal women. However, its relationship with lung cancer remains controversial. Another study found that combined estrogen and progestin therapy failed to increase lung cancer incidence in postmenopausal women. However, it was associated with more lung cancer‐related deaths, especially from non‐small cell lung cancer [147]. These findings should inform risk–benefit discussions with women considering combined hormone therapy, particularly those at higher risk for lung cancer.

Taken together, current evidence shows that lung cancer risk differs between women and men. Women seem more susceptible to tobacco carcinogens, likely due to hormonal and other environmental factors. Therefore, sex should be considered an independent factor in prognosis and treatment decisions.

### 3.2 Sex Differences in Susceptibility to Lung Infections (Figure 3)

The role of biological sex in the severity of respiratory infections is widely documented in both murine models and in human subjects. Numerous studies have highlighted the role of sex hormones in regulating immune mechanisms to infection via the action of estrogen receptor, progesterone receptor, and androgen receptor in immune cells. Precisely how sex hormones promote resolution of infection remains unclear. Here, we review sex‐biased immune responses to common bacterial and viral infections in the respiratory tract.

#### 3.2.1 Bacterial Infections

In a mouse model of pneumococcal infection investigating sex hormone regulation of immune responses, male mice exhibited a cytokine profile consistent with severe neutrophilic inflammation [148]. Proinflammatory cytokines IL‐6, IL‐1β, IL‐6, and IL‐17A, KC and MIP‐2 (neutrophil chemoattractant) and M‐CSF, MCP‐1 and GM‐CSF (the activator/proliferator of monocytes, macrophages) were all significantly higher in the airways of male mice at 24h [148]. The authors found that male mice succumbed to lung infection and associated pathology at a significantly higher rate, even when challenged with a10‐fold lower pneumococcal dose, showing a similar 6‐h increase in pneumococcal cfu. This sustained increase in proinflammatory mediators caused significant lung pathology in males, irrespective of increasing bacterial dose and was consistent in respiratory infection and systemic disease [148]. Female mice, on the other hand, which produce lower amounts of proinflammatory cytokines, presented with reduced neutrophil recruitment after 24h and survived the infection despite harboring a similar pneumococcal burden in their lungs as male mice after 24h.

Conversely, following Pseudomonas Aeroginosa infection in a mouse model of cystic fibrosis, females were found to be more susceptible to infection and died earlier, showing slower bacterial clearance than males [119]. This phenotype was rescued in ovariectomized mice. Furthermore, administration of 17β‐estradiol to ovariectomized mice increased expression of the inflammatory cytokines IL‐6, TNF‐α, MIP 1α and MCP‐1 in the lungs of infected mice, accompanied by neutrophil dysfunction and a worse outcome than in infected male mice and vehicle‐treated female mice. This was found to be ER dependent, whereby antagonism of ER signaling improved survival in female mice infected with *P. aeruginosa* and restored neutrophil function [119].

In mouse models of tuberculosis, *Mycobacterium tuberculosis* infection caused significant mortality in male mice compared to female mice. Male mice' lungs harbored higher numbers of bacterial colony–forming units (cfu). This was associated with reduced pro‐inflammatory responses in male mice, with lower macrophage and lymphocyte counts and lower production of pro‐inflammatory cytokines compared to female or castrated mice [149]. Moreover, administration of medroxyprogesterone acetate (DMPA) decreased the *M. tuberculosis* induced cytokine production in C57BL/6 and BALB/c mice [150].

Furthermore, in humans following tuberculosis infection, men display higher levels of platelet‐derived growth factor subunit β (PDGFβ), serum antibodies against *M. tuberculosis* , and serum C‐reactive protein while women display higher concentrations of the C‐X‐C Motif Chemokine Ligand 9 (CXCL9), while men show higher levels of the suggesting that males exhibit a stronger innate and humoral immune response to tuberculosis than females [148]. Interestingly, unvaccinated males, when challenged with *Bacillus Calmette‐Guerin (BCG)*, display a higher IFN‐γ response against the tuberculin purified protein derivative (PPD) than females [149]. This suggests that males present with an uncontrolled pro‐inflammatory response and a greater susceptibility to *M. tuberculosis* infection [149].

#### 3.2.2 Respiratory Viruses

Respiratory viruses are well documented to breach the structural and biochemical barrier that protect the host. Viruses inflict damage either directly by productively infecting lung cells or indirectly by generating strong pulmonary inflammatory immune responses. Upon respiratory virus infection, immune cells recognize viral particles and initiate early anti‐viral responses to prime the adaptive response. This provokes adaptive immune cells to clear virus‐infected cells and produce virus‐specific antibodies and generate memory T cells [150]. Finally, tissue regeneration mechanisms are initiated involving multiple immune cells, structural cells, and mediators to promote lung regeneration and immune homeostasis [150].

##### 3.2.2.1 Influenza A Virus (IAV) Infection

Men exhibit a higher incidence of infection with influenza, but females usually experience greater morbidity. This increased severity of the infection in females results from a stronger innate and adaptive immune response, which leads to enhanced immunopathology. Epidemiological data from multiple flu outbreaks show that hospitalization rates and mortality among females are compared to those of males during their reproductive years [151]. These findings suggest that adult sex hormone levels modulate immune responses to IAV, but the molecular or cellular basis of these immune responses remains poorly defined. Females of reproductive age also have a higher incidence of asthma [2] which may influence immune responses to IAV and worsen IAV‐induced pathology. In contrast, infection in young males (under 20 years) and elderly adults (over 80 years) led to more hospitalizations or deaths [84]. This pattern may suggest that low levels of androgen in young boys and elderly men are linked to worse infection outcomes. However, more data on comorbidities, direct androgen measurement, and male susceptibility before and after puberty are needed to confirm this relationship. Sex‐based differences in susceptibility and immune responses to influenza A virus (IAV) were observed in mice infected with mouse‐adapted H1N1, avian H3N1, and H7N9 viral strains. Female mice exhibit greater morbidity, mortality, and inflammatory responses than males at moderate IAV loads, although mortality rates converge at higher viral loads [152, 153]. At sublethal doses, female mice produce more CCL2, IFNγ and TNFα as well as increased antibody titters, which are associated with enhanced protection during heterosubtypic virus challenge [153]. When exposed to viral doses lethal for females but not males, estrogen confers protection against mortality, as demonstrated by studies comparing ovariectomized mice treated with estradiol or placebo [152]. Estrogen supplementation reduced the production of TNFα and CCL2 and increased neutrophil counts and increased IAV‐specific CD8^+^ T cells that were capable of producing IFNγ [154]. Collectively, both females mice with intact gonads and gonadectomized female mice generate stronger inflammatory responses and experience heightened morbidity following infection, indicating that low estrogen levels may drive excessive inflammation. Conversely, administration of higher estradiol levels in gonadectomized mice reduces inflammation and protects mice from infection. These data may explain why children are highly susceptible to influenza infections and can experience severe disease presentation due to reduced pre‐existing immunity. Age, concomitant with sex‐based differences in immunity, significantly contributes to susceptibility. One study found females younger than 20 or > 80 had lower morbidity rates during the 2009 influenza pandemic compared with males [155].

Juvenile female mice infected with influenza maintained a broader cytokine response in the lung following clearance of influenza, marked by innate, type I and type II cytokine production [156]. In male juveniles, higher levels of IL‐6 and other macrophage‐related cytokines were detected, but there was no evidence of a type I or type II response. Additionally, male mice exhibited a higher regulatory T cell to T_H_1 ratio compared with female mice. These data suggested that following IAV infection, juvenile female mice have persistent and display a more immunosuppressive phenotype [156].

Ovariectomy in female subjects, followed by replacement of progesterone to luteal phase concentrations, reduced IAV‐induced morbidity following infection. Progesterone enhanced tissue repair by upregulating the epidermal growth factor receptor ligand amphiregulin (Areg) in the lung [157]. Collectively, these findings indicate that progesterone‐based contraceptives may facilitate recovery from respiratory virus infections.

Gonadectomy in young male subjects increased morbidity and pathology following IAV infection, whereas replacement with testosterone or dihydrotestosterone (DHT), which is not metabolized to estradiol, reduced morbidity, mortality, and inflammation. In contrast, testosterone administration in aged male mice, which exhibit reduced testosterone levels, improved survival but lung pathology remained unchanged [158].

##### 3.2.2.2 Respiratory Syncytial Virus (RSV) Infection

RSV infection is more common in young boys, however the precise underlying mechanisms governing this sex difference remain unclear. Previous research highlights the importance of sex hormones in regulating neutrophil function after viral infection. Neutrophils produce a range of pro‐inflammatory cytokines and reactive oxygen species (ROS) [159]. However, their precise role is not fully understood, since they can also cause tissue damage and increase the likelihood of secondary infections. The number of neutrophils and the formation of neutrophil extracellular traps (NETs) are directly linked to the severity of RSV infection [160].

Studies in mice show that neutrophil numbers are regulated by sex hormones during both at homeostasis and after infection. Mice deficient in androgen receptors (AR) exhibit reduced neutrophil numbers and neutrophil precursors in bone marrow (BM) [161]. In contrast treating ovariectomized female mice with estradiol increases neutrophil chemoattractant levels and recruitment of neutrophils to the lungs, thereby enhancing protection during influenza A virus (IAV) infection [154].

Neutrophils are known to express both estrogen and androgen receptors, and in humans, the number and function of blood neutrophils differ between females and males. In humans, blood neutrophil counts go up during pregnancy, suggesting that elevated progesterone or estrogen levels may promote neutrophil differentiation and maturation [162]. Neutrophils from young women of reproductive age survive longer in lab tests than those from healthy men. Estradiol and progesterone have been shown to delay neutrophil cell death by reducing caspase 3 activity, which promotes apoptosis [163]. Other research shows that sex hormones can affect neutrophil function by regulating their production of nitric oxide and superoxide [164, 165].

Eosinophils also promote anti‐viral immune responses after RSV infection by sensing viral RNA via TLR7, thereby inducing nitric oxide production [166]. Eosinophils were found to degranulate efficiently and activate virus‐specific CD8^+^ T cells, which increase protection against infection [167].

Taken together, female sex hormones may regulate eosinophil numbers; however, further research is warranted to precisely understand how sex hormones control lung eosinophil numbers and function during RSV infection.

##### 3.2.2.3 Severe Acute Respiratory Syndrome Coronavirus 2 (SARS‐CoV‐2) infection

The Coronavirus Disease‐2019 (COVID‐19) resulted in a global pandemic that affected millions of people worldwide. In the year 2021, 100 million confirmed cases of COVID‐19 were reported, among which 2 million deaths were reported worldwide. The Severe acute respiratory syndrome coronavirus 2 (SARS‐CoV‐2) causes COVID‐19 and has a complex interaction with the immune system. SARS‐CoV‐2 infection resulted in a “cytokine storm syndrome” and was accompanied by increased innate immune responses, overproduction of pro‐inflammatory cytokines, uncontrollable inflammation leading to multiple‐organ failure and death in infected individuals. SARS‐CoV‐2 also impaired adaptive immune responses, resulting in excessive tissue pathology. A growing body of evidence suggests that sex‐specific differences in the immune response to SARS‐CoV‐2 exist with males exhibiting higher disease severity and males are almost three times more likely to require intensive treatment unit (ITU) admission compared to females and are at a higher risk of succumbing to the disease [168].

Studies by Takahashi et al. investigated sex differences in viral loads, virus‐specific antibody titres, plasma cytokines and blood leucocyte immunophenotyping in patients with moderate COVID‐19 who had not received immunomodulatory medications [169].

The authors performed baseline and longitudinal analyses and showed that males had higher levels of plasma cytokines, including IL‐8 and IL‐18 (baseline) and stronger induction of non‐classical monocytes and CCL5 (longitudinal), compared to females monocytes (at baseline) [169]. Moreover, females infected with SARS CoV2 had robust CD8 T cell responses compared to male counterparts and healthy volunteers. These immunological changes governed how the infection progressed between females and males during acute SARS‐CoV‐2 infection.

Similar to acute infection, sex differences were also observed in long COVID (LC). In contrast to males having greater disease severity and mortality during acute infection, a larger number of females developed LC [170]. To interrogate the immunologic pathways involved in LC development and symptom persistence, the authors performed multi‐omic analyses on infected patient blood samples at 3 and 12 months after infection among a patient group that had developed LC or recovered from infection. Immunological analysis revealed that males who would go on to develop LC had increased serum transforming growth factor–β (TGF‐β) expression in acute infection, whereas female counterparts that developed LC had reduced TGFβ1 expression in acute infection [170]. Female patients experiencing LC disease displayed increased expression of XIST, an RNA gene implicated in autoimmunity, during acute infection compared with females who recovered [170]. Other immunological changes due to infection, for example, alterations in monocyte phenotype, monocyte activation, and upregulation of nuclear factor κB (NF‐κB) transcription factors were comparable between the sexes. LC patients also presented with reduced ETS1 expression across lymphocyte subsets and elevated intracellular IL‐4 in T cell subsets, suggesting that ETS1 alterations may drive aberrantly elevated T helper cell 2–like responses in LC. Therefore, sex regulated some but not all immunological changes between sexes and set the foundation for developing therapeutic strategies against LC [170].

A growing body of evidence suggests that gonadal sex hormones influence sex differences in COVID‐19 progression.

A study by Yeap et al. quantified testosterone concentration in men many years before SARS CoV‐2 exposure and found a direct correlation between serum testosterone concentrations and the risk of death from COVID‐19 [171]. Similarly Montopoli et al. investigating cohort multicentre studies found that exogenous lowering of testosterone concentrations by androgen suppression therapy (ADT) supported the recovery of patients from severe COVID‐19 disease [172]. In a large retrospective study involving female COVID‐19 patients, an association between sex hormones, menstrual status, and immune‐inflammation‐related cytokines and COVID‐19 disease prognosis was carried out [173]. The authors found that E2 and anti‐Müllerian hormone (AMH) were negatively associated with the severity of COVID‐19 infection [173]. Therefore, E2 and AMH may serve a protective role in COVID‐19 infection. Furthermore, E2 was found to interfere with the interactions between the human Angiotensin‐converting enzyme 2 (ACE2) and the SARS‐CoV‐2 entry receptor, thereby blocking viral entry into cells [174]. Despite the involvement of sex hormones in COVID‐19 pathogenesis, further research is needed to comprehensively explore the impact of sex hormones across different strains of SARS‐CoV‐2 and to identify the precise mechanisms involved in these interactions.

Overall, the literature suggests that stronger antipathogen responses are tissue and context specific and, in most cases, females may be evolutionarily tuned to be protected through their childbearing and rearing years at the expense of autoimmune disease later in life, and functional adaptation to these local demands likely contributes to sex‐ and tissue‐specific immune phenotypes.

## 4 Concluding Remarks

The relationship between sex and lung immunity is complex but significantly affects the pathophysiology of common lung diseases. It is vital that we understand how sex hormones, as well as sex chromosomes, affect the course of pulmonary diseases across the lifespan. This is particularly important given the advent of biological agents designed to target key immune cells and cytokines; we need to understand how these agents may work differently in males versus females. Addressing these challenges necessitates the selection of appropriate pre‐clinical models with balanced physiological relevance while maintaining the capacity to test specific hypotheses. Murine models described in this review serve as essential resources for investigating hormonal and chromosomal contributions to the immune system and will help generate insights that bridge the gap between animal studies and human applications.

Going forward, we need to ensure that all future studies in both mice and humans factor in biological sex as an essential immunological variable by incorporating this additional level of stringency into experimental design, interpretation and reporting in both human and animal studies. It may also be valuable to plan human immunological studies that include individuals undergoing sex hormone replacement therapy to better understand how artificial administration of sex hormones directly impacts immune function with respect to pulmonary health. Addressing these challenges necessitates the selection of appropriate pre‐clinical models, with balanced physiological relevance while maintaining the capacity to test specific hypotheses. Murine models described in this review serve as essential resources for investigating hormonal and chromosomal contributions to the immune system and help generate insights that bridge the gap between animal studies and human applications.

Emerging technologies, including in vitro organoid systems combined with computational modeling and multi‐omic analyses, will help uncover new molecular mechanisms underlying sex differences in lung immune responses and aging. Furthermore, incorporating sex as a variable in clinical trials in multiple lung conditions, particularly those evaluating vaccines and immunotherapies, is essential for translating pre‐clinical discoveries into personalized interventions.

In conclusion, understanding sex‐specific immune responses is fundamental for advancing both biological research and personalized medicine. Prioritizing sex as a core variable and adopting innovative research models will facilitate the translation of findings into effective therapies and improve health outcomes.

## Funding

This work was supported by Wellcome Trust, 107059/Z/15/Z, 220254/Z/20/Z. Asthma and Lung UK, ECSG24\26.

## Conflicts of Interest

The authors declare no conflicts of interest.

## Data Availability

The data that support the findings of this study are available from the corresponding author upon request.
